# Advancements in Surface Acoustic Wave Gyroscope Technology in 2015–2024

**DOI:** 10.3390/s25030877

**Published:** 2025-01-31

**Authors:** Alexander Kukaev, Egor Shalymov, Sergey Shevchenko, Maria Sorvina, Vladimir Venediktov

**Affiliations:** 1 Department of Applied Mechanics and Engineering Graphics, Saint Petersburg Electrotechnical University, 197376 St. Petersburg, Russia; 2 Department of Laser Measurement and Navigation Systems, Saint Petersburg Electrotechnical University, 199034 St. Petersburg, Russia; shev1989@yandex.ru (E.S.); vlad.venediktov@mail.ru (V.V.); 3 Quantum Electronics Department, Faculty of Physics, Saint Petersburg State University, 199034 St. Petersburg, Russia

**Keywords:** surface acoustic waves, gyroscope, inertial navigation, parameter optimization, acousto-optic gyroscope, phononic crystal, FEM simulation

## Abstract

Although the theoretical basis for surface acoustic wave gyroscopes (SAWGs) was first proposed in 1980, their design concepts are still under development. Nevertheless, these sensors are of a great interest in the potential market owing to their exceptional shock resistance, small size, low power consumption, and simple manufacturing process that ensures low cost. This paper aims to conscientiously investigate the ideas that have been proposed over the past decade in this area and evaluate the potential development required to bring SAWGs to market. It should be of interest for researchers in the field who might have missed some useful solutions that could be a missing piece in their own design, or for young researchers to inspire their creativity and open new research on the topic. Additionally, since some of the reviewed SAWG design concepts are based on a combination of several physical principles (for example, optical measurements), researchers from other fields may find useful solutions for incorporating surface acoustic wave techniques into their device concepts.

## 1. Introduction

In recent decades, sensors based on surface acoustic waves (SAWs) have not only strengthened their positions in existing applications, but also significantly expanded their areas of utilization. Compared to MEMS gyros, which today occupy the area of mass-market inertial sensors, SAW-based gyros (SAWGs) ultimately provide better shock and vibration resistance due to not having any moving parts or vulnerable suspensions. At the same time, their fabrication process is even more simple and, therefore, the SAWG mass-production price should be at least competitive, or even much lower. Thus, owing to its low cost, small size and weight, outstanding shock resistance, and possibility of wireless and passive operation, such sensors are of a great interest in many industries. This fact motivates different research groups to further investigate possible design implementations and search for new concepts. Significant work has been performed by scientists from different countries dedicated to the development of SAW-based inertial sensors, i.e., gyroscopes and accelerometers.

These types of sensors are more complicated in their structure than temperature, torque, or gas sensors. Their parameters strongly depend on the wafer material, geometry of the sensing element, and the electrical scheme parameters. However, the most challenging task for the researchers is probably to find the proper topology of electrodes and other deposited structures (reflectors, inertial masses, buses, heaters, etc.) that provides the best performance of the sensor. A good topology not only isolates the measured parameter from all the others, but also maximizes the sensitivity, keeps the size of the sensor sensible, ensures easy and cheap production, etc. Some of these goals might be achieved by combining already known elements in a specific way, while others require the proposition of novel solutions. In this case, some preliminary calculations or simulations are required to check the vitality of the proposed idea and evaluate potential sensors characteristics.

A previous comprehensive review of SAW-based gyroscopes was published by Oh et al. in 2015 [1]. Despite its short length, it clearly depicted two basic sensing principles (on running and standing waves), addressed major existing design concepts, and highlighted a couple of pioneering ideas. However, almost a decade has passed since that review, and a lot of work has been carried out by the worldwide scientific community to bring closer the day when an affective and competitive SAWG will enter the market of compact inertial sensors. The scope of this paper is to systematize the research on the topic of SAWG made since 2015. The main purpose is not only to state the major tendencies, but also to highlight some brave pioneering ideas that might help overcome some specific problems.

Before passing to the main point of this paper, let us underline some principles used to choose the papers included in the review. As was already mentioned, we are not addressing papers dated earlier then 2015 unless necessary according to the section context. Although we tried our best to explore all the papers on the specified topic during the stated period, we understand that some might have remained hidden from our sight, or were left unread due to being written in a local language unfamiliar to the authors.

Also, we understand that SAW-based sensors of different physical parameters, SAW-filters, and other devices are also being developed and some solutions found might be utilized in the construction of SAWGs. However, it is not really possible to combine all the ideas in the area of SAW devices in one paper, even limiting them to those useful for SAWGs. Specifically, the topic of SAW-based accelerometers, which are also sensors of the inertial kind and in many cases are developed by the same research groups, is hoped to become the topic of a separate review paper.

Despite the fact that a comprehensive paper dedicated to the topic of SAWGs may contain a theoretical explanation, simulation part, and experimental results, any of which may describe a novel design approach, we decided to separate the papers under review into the following groups:

1. Theoretical works that contain only theory, no simulation (except numerical evaluation of obtained expressions) and no experiments.
2. Works dedicated mainly to simulation and modeling. These papers may contain some novel construction ideas and corresponding theoretical findings, but do not have any experimental evaluations.
3. Works describing the optimization of well-known design principles. These papers may contain a theoretical part, numerical modeling, and experimental results, but are primarily dedicated to improving design concepts that do not differ much from well-known examples.
4. An overview of papers that describe totally new ideas, for example, incorporating other physical principles, and are illustrations of “outside-the-box thinking” is completed in Section 6.
5. The final section that precedes the Discussion Section is dedicated to the papers describing novel ideas in the SAWG fabrication process.

## 2. Basic Operation Principles

Assuming that not all the readers might be familiar with the topic, let us shortly describe very basic operation principles of SAWGs. The classic design concepts utilize two fundamental physical effects: the piezo effect to generate and detect surface acoustic waves and the Coriolis effect that makes these waves vulnerable to the rotation of the sound conductor. The latter may be used in two different ways, depending on the type of the wave used in the particular device: a running or a standing one. The selected wave type also determines the type of a transducer to be used—a delay line for running waves, or a resonator to form the standing one. The theory of SAW propagation in a rotated medium is well known [2,3,4,5,6], so we will only illustrate shortly how this theory works in a real device.

### 2.1. The Coriolis Effect in Running SAWs

**Figure 1:**
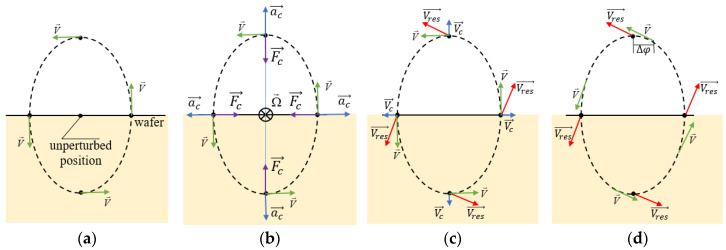
Coriolis effect in running SAWs. (**a**) Particle velocities in unperturbed wave, (**b**) diagram of Coriolis forces when subjected to rotation, (**c**) directions of resulting particle velocities, (**d**) demonstration of the phase shift caused by Coriolis force.

We consider a piezoelectric crystal as a structured system of molecules that vibrate around their unperturbed position when a SAW is running through it. We will use Rayleigh waves as an example since waves of other types (Love waves, for example) demonstrate the same effects, with only the axes orientation changed. It is known that in this case surface particles move along elliptical trajectories and, therefore, they have a linear velocity *V* (Figure 1a), which has a different direction in all trajectory points, but obviously is always tangent to it.

When the wafer with a running SAW is rotated around the axis that lies in its surface and is orthogonal to the particle moving plane, the Coriolis acceleration *a_C_* and the corresponding force *F_C_* start their action (Figure 1b). The interaction between the original movement components and the ones induced by the Coriolis force has different interpretations [2,3,7]. Without going deep into the theory, we will give the simplest one. Let us consider the geometrical sum of the particle velocity vector induced by the Coriolis force and the one induced by the original running SAW (*V_res_* in Figure 1c). The resulting vector is slightly rotated compared to the original one as shown in the figure. However, the whole picture of new velocity directions matches the picture of unperturbed velocity directions with a slight phase shift (Figure 1d). So, the rotation of the wafer produces a phase shift in the running wave, which is proportional to the applied rotation rate. Due to the wave nature, it will not be a mistake to interpret this as a change in the wave phase velocity. While the phase shift might be measured directly, it is usually more convenient to convert it into the change in the frequency of the oscillator which utilizes the delay line of the running wave under consideration. In this case the change in the phase shift caused by the DL results in the violation of the phase-balance inside the oscillator and, consequently, the shift of its operating frequency.

### 2.2. The Coriolis Effect in Standing SAWs

**Figure 2:**
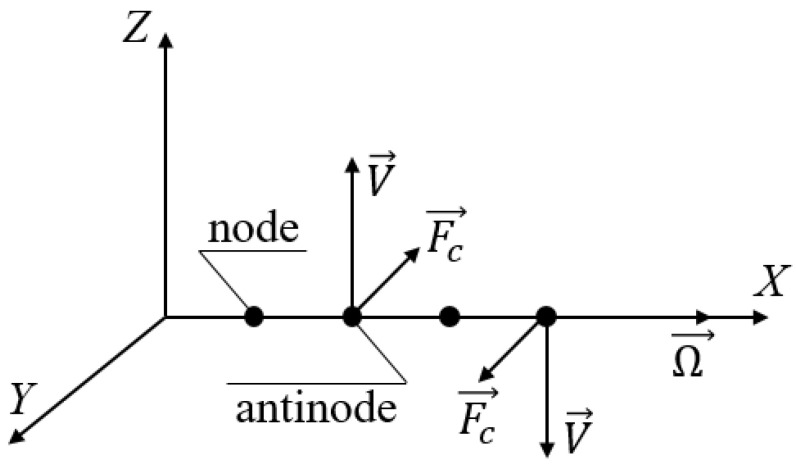
The Coriolis effect in standing SAWs.

While this effect is comparatively easier to explain, it usually requires more complicated electrode topologies to be utilized. In standing waves particle trajectories are much more stretched out in comparison to running ones, so it is possible to simplify them to straight lines orthogonal to the wafer surface. Also, in this case only antinodes have linear velocities, while particles in wave nodes rest in their unperturbed positions. Let us consider a particle in the antinode that has the original linear velocity *V* and is subjected to the rotation velocity Ω, which in its turn causes the Coriolis force *F_C_* (Figure 2).

As can be seen, in this case the Coriolis force and the corresponding particle movement component is oriented orthogonal to the original particle movement plane. Due to the constant direction change, the Coriolis force acting on the entire particle ensemble generates a running wave propagating orthogonally to the original SAW. The amplitude of the induced wave is proportional to the rotation velocity. The mechanics of how to estimate the amplitude of this wave differs in particular implementations. The simplest way is to put an interdigitated transducer (IDT) to transform it into an electrical signal and after that measure its level directly. Some more specific approaches will be considered further in this paper.

The above-described effects explain how the rotation velocity acts on the SAW and “loads” the information about its value into the wave parameters. However, surface acoustic waves are vulnerable to many other external factors, such as acceleration, temperature, humidity, pressure, and torque. For this reason, a lot of research is carried out not on the problem of how to make the SAW device sense something, but on how to prevent it from sensing everything else. Another typical problem is insufficient sensitivity caused by the weakness of the above effects. It originates mainly from the negligible mass of moving particles. And, finally, some specific applications (wireless and passive operation, for example) require more peculiar solutions. Before discussing novel design concepts, let us give an overview of progress in theoretical approaches.

## 3. Theoretical Works

The first theoretical description of a SAW travelling in a rotating medium was given by Lao in 1980 [2]. Further research extended these results for different materials (especially anisotropic ones) and wave polarizations (Rayleigh, Love, etc.). However, some specific cases are still being found and considered in publications.

For example, in 2019 Hu et al. described the gyro effect on SAW propagation in layered structures [8]. Based on the model of a sound conductor made of a thin piezoelectric film deposited on the elastic halfspace subjected to rotation, the authors made two conclusions:

1. Long waves are more sensitive to rotation than short ones;
2. A more evident change in frequency occurs when the free surface is electrically shorted.

The first conclusion is similar to the well-known fact that SAWs with a lower frequency exhibit a larger gyroscopic effect. This principle was predicted from the pioneering work by Lao. His final formula was later rewritten in a more convenient form by Lee [3]:

$$\frac{\Delta V}{V}=\beta \frac{\Omega }{\omega },$$

where *V* is the initial wave propagation velocity, Δ*V* is its change due to the rotation rate Ω, *β* is the scale coefficient that depends on the material properties, and ω is the wave angular frequency. If we consider this as the Δ*V*(ω) dependence, it is quite obvious that lower frequencies provide a stronger effect.

One more theoretical work on the topic was published in 2023 by Yuan et al. [9]. The authors investigate the case of a Rayleigh wave propagation under the effect of rotation in an electrical biasing field. The results obtained are quite arguable. The main achievement stated by the authors is that at high rotation rates the wave velocity becomes much lower, which means that the IDT finger width and spacing might be reduced, resulting in the miniaturization of the whole sensor. For example, according to the paper, at the rotation rate of 10^6^ rad/s (which is already a ludicrous value) the wave velocity should lower from approx. 4500 m/s to 500 m/s, which gives the opportunity to make a sensor up to 10 times smaller.

Without getting into discussion about the physical feasibility of such a dramatic change in velocity, let us just remark that, first, the IDT parameters are chosen in accordance with the wavelength rather than wave velocity, and, second, if we adapt the IDT parameters to the circumstances that appear only within a certain rotation rate range, the sensor will work correctly only in those circumstances and will be useless in practical terms.

Nevertheless, the idea of investigating the effect of biasing electric field acting on a rotated sound conductor looks promising under some specific conditions. In 2024, Yang et al. published their results describing the rotation effects on SAW propagation in piezomagnetic-piezoelectric semiconductor layered structures [10]. The paper presents a deep study on the problem of a shear-horizontal wave propagating in a thin piezoelectric semiconductor film deposited on the piezomagnetic substrate subjected to rotation in a biasing electromagnetic field. In the numerical evaluation part, the authors made calculations assuming that the piezomagnetic substrate is made of CoFe_2_O_4_, and the piezoelectric film is ZnO. The results showed that in this case not only will the wave speed decrease with the increase in the rotation rate, and this effect will be higher with lower SAW frequencies (which is a well-known fact for common design concepts), but also that:

1. The wave attenuation also decreases with higher rotation rates.
2. The biasing electric field, along with the steady-state carrier concentration, has a strong influence on the wave velocity and attenuation; these dependencies have extremum points and might be used as an instrument for SAWG parameters optimization.

Even though the paper does not provide a sensor design, the proposed ideas seem quite promising, and the paper might have been included into the “New physical principles” section (Section 6 in this paper) if it were not purely theoretical.

## 4. Simulation and Modeling

**Figure 3:**
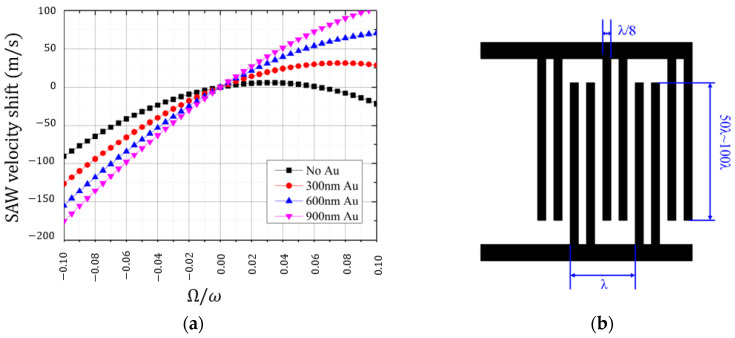
Impact of the electrode thickness on the gyroscopic effect in an IDT (**a**), and a split IDT structure (**b**) [11].

In 2019, Sun et al. published an article concerning the investigation of the SAW gyroscopic effect in an IDT [11]. Utilizing coupling-of-modes (COM) and two-dimensional finite element (FEM) methods, the authors investigated the impact of metal electrode thickness and the IDT structure on the value of a gyroscopic SAW velocity shift. The sensor structure was not their point of interest and, probably, the results are suitable for all sensors incorporating the gyroscopic effect on running SAWs (see Section 2.1). Both modeling approaches were in a good agreement, and the simulation showed that thicker metallization (at least in the span of 0–900 nm) provides a greater gyroscopic effect (Figure 3a). However, the main finding of the paper was that the reflectivity of the IDT structure is the characteristic that has a strong impact on the device sensitivity. Using different electrode structures (i.e., classic IDT, single phase unidirectional transducer (SPUDT) and split IDT) that have different reflection coefficients, the authors obtained a SAW frequency shift of approx. 698 Hz for the most reflective bidirectional IDT and 36 kHz for the least reflective split IDT (Figure 3b). So, the latter should be considered as the most effective unless the specific design concept requires the IDT to be unidirectional.

In 2022, almost the same group of authors proposed an idea to utilize a layered structure of 128° YX LiNbO_3_/SiO_2_/Si substrate [12]. Since the SAW device is vulnerable to many physical quantities, the problem of choosing a proper material has always been of immediate interest for SAWGs. A typical solution was to maximize the electromechanical coupling coefficient *K*^2^ for better sensitivity and neglect the high temperature coefficient of frequency (TCF), which might be achieved by utilizing the differential measurement scheme. From this point of view, lithium niobate was the most promising and, therefore, the most frequently chosen material. In cases when the differential scheme was impossible or ineffective, the best option was to use ST-cut quartz, which has a TCF around zero. However, the paper [12] provides an interesting solution that makes it possible if not to eliminate all the material problems, then at least to have an instrument to adjust parameters to find some optimal solution. In the above-mentioned layered structure, materials are combined in such a manner that their virtues and shortcomings compensate one another. So, the TCF, *K*^2^, gyroscopic gain factor, and SAW velocity of the entire substrate depend on the ratio of the layers thicknesses and might be adjusted to meet the needs of a particular application. The obtained results are mainly supported by the FEM simulation in COMSOL and it would be of great interest to see their experimental verification. Nevertheless, the idea is clear and offers a good instrument for researchers.

Also, in 2022, Wang et al. published their work dedicated to the optimization of inertial masses utilized in the structure of a running SAW-based gyro [13]. This paper might have been discussed in the “Optimization” section (Section 5 of this paper) if, apart from a simulation, it had some experimental results.

**Figure 4:**
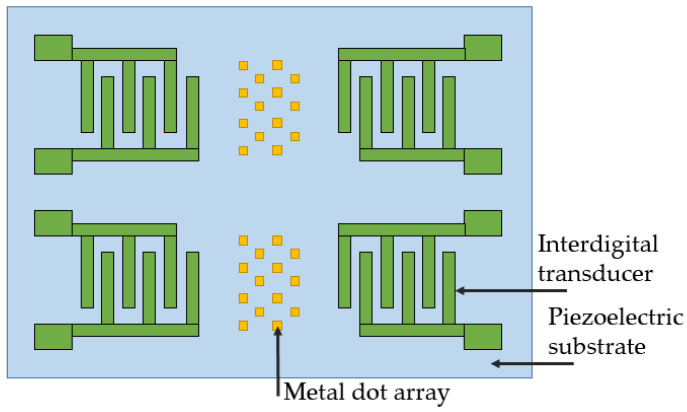
Structure of the sensor considered in [13].

The paper develops the idea previously proposed by the same authors [14]. Here, they propose to deposit a matrix of small inertial masses to the free space of the SAW delay line to enhance the Coriolis effect (Figure 4). Since the Coriolis force is proportional to the mass of a vibrating particle, its increase should enhance the effect, thus providing a better sensitivity. However, depositing a metal layer over the sound conductor has some additional effects, such as changing the wave propagation conditions, changing the overall stiffness in the deposition region, and making it more difficult for the particles to vibrate under the weight of the mass. So, it is important to find an optimal material and size of the inertial mass for it to have a positive effect. This is what was stated as the goal of the paper [13]. The authors made a 3D FEM simulation of a metallic dot on a wafer subjected to rotation. The materials they considered form “the Holy Trinity” of materials used in SAW-based sensors: 128° YX LiNbO_3_ (as a material with the highest *K*^2^), ST-cut quartz (as a material with the best TCF), and X112°Y-LiTaO_3_ (as a compromise between the previous two). The results show that for each material there is an optimal value of the inertial mass size, which is 3λ/16 for ST-cut quartz, 5λ/16 for 128° YX LiNbO_3_, and λ/16 for X112°Y-LiTaO_3_, where λ is the wavelength. The most surprising result is that when utilizing the optimal inertial mass, ST-cut quartz provides the best performance in terms of the gyroscopic gain factor. Saying that, Wang et al. emphasize that the specific reasons for this are not clear and require more in-depth study.

The above-mentioned fact makes the next paper published by the same authors [15] even more interesting. Here, they develop the same idea of using the optimal-sized inertial masses but, in some sense, combine it with ideas proposed in the previously mentioned paper by Ma et al. [12]. The idea is to use the same measurement principle and the same electrode and inertial mass topology, but to utilize a layered substrate consisting of a thin piezoelectric film deposited on a non-piezoelectric substrate. Si, SiC, and diamond are considered as a substrate. Using the same research apparatus, the authors come to the following conclusions:

1. In a double-layer substrate structure, the greater the difference in reflection coefficients between the upper and lower-layer materials, the higher the sensor sensitivity is. From this point of view, the diamond used as the lower layer shows the best performance.
2. Reducing the piezoelectric film thickness enhances the sensor sensitivity as well.
3. The optimal size of the metallic dot (the inertial mass) should be calculated separately for each substrate structure.

Unfortunately, no specific comments were added on the topic of the outstanding ST-cut quartz performance, mentioned in the previous work, even though the combination of ST-cut quartz on the diamond was once again found to be the best choice. In any case, the work carried out by the group of prof. Wang presented in these papers is definitely remarkable and of a great interest for further investigation.

## 5. Optimization

In 2015, a well-known group of authors leaded by prof. Wang published results of their work [16]. This paper chronologically stands between the two above-mentioned works presenting their idea [14] in 2014 and a further simulation approach in 2022 [13]. Here, the main focus was on the optimization of materials of both the wafer and the metallic dots, inertial mass thickness, and the device operation frequency. The results of preliminary theoretical calculations were supported by further experimental verifications. Main findings are the following:

1. In terms of the wafer material, LiNbO_3_ shows better performance than LiTaO_3_, probably because of higher *K*^2^.
2. Inertial masses made of gold show higher sensitivity than those made of copper, and devices with inertial masses are definitely more sensitive than those without inertial masses at all.
3. Thicker metallic dots also provide better sensitivity.
4. Lower operation frequency is accompanied by better sensitivity.

In general, the results just prove the idea that an increase in the mass subjected to vibration and, consequently, to the Coriolis effect provides an increase in the device sensitivity. This final conclusion about the frequency has already been mentioned in several works reviewed above. Nevertheless, the experimental proof of these ideas was an important milestone. Finally, it is worth mentioning that the device with the optimal combination of parameters showed the scale factor of 43 Hz/(°/s) with almost zero temperature dependence.

In 2017, a group of Russian researchers presented their approach to the optimization of the form of inertial masses utilized in standing waves based SAWGs [17]. Unlike conventional λ/4 × λ/4 size masses, here the authors proposed to use λ/2 × *A* masses, where *A* is the SAW aperture. Additionally, the flank edges should have a triangular shape instead of being flat to provide force concentration. All these novelties were designed to further increase the Coriolis force acting on the vibrating masses. Due to the lack of experimental results, the authors only presented their theoretical predictions of a possible gain. However, their work did not go any further.

**Figure 5:**
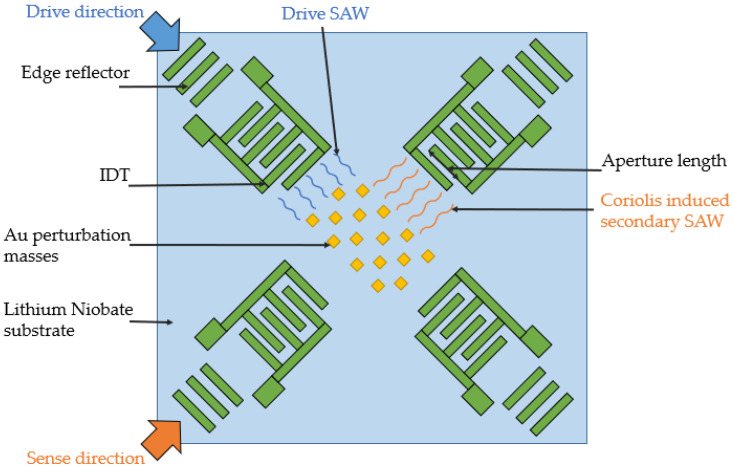
The scheme of the orthogonal 45° rotated SAWG described in [18].

Though the idea of a SAWG based on standing waves receives a lot of criticism due to their low sensitivity, high complexity and lack of thermal stability, a group consisting of Ashraf Mahmoud, Mohamed Mahmoud, Tamal Mukherjee, and Gianluca Piazza have made a big effort to bring this idea to life. During 2018–2020, they published 4 papers, all dedicated to the improvement of the same device [18,19,20,21] (Figure 5). Its working principle is the same as in the first concept proposed by Kurosawa in 1997 [22]. Here, a standing SAW generated in the resonator oriented in the drive direction exhibits the Coriolis effect the way it is described in Section 2.2. The SAW generated due to this effect propagates to both sides in the sense direction and serves as the informative signal utilized by the corresponding sense resonator. A matrix of small metallic dots (inertial masses) is deposited in the intersection of drive and sense resonators to magnify the Coriolis effect.

The first idea proposed by Mahmoud et al. was to rotate the resonator by ±45° relative to the *Y*-axis, to ensure frequency alignment between the driving and sensing resonators despite the anisotropic nature of the wafer material (Y-cut Lithium Niobate) [18]. Next, they fabricated two devices that had different aperture lengths, number of IDT fingers and inertial mass thickness. The best of these two showed the scale factor of 0.959 µV/(°/s). It is worth noting that this paper was, probably, the first to provide the value of angle random walk of a real SAWG device, which was 5.95${}^{\circ}/\sqrt{h}$. The theoretical prediction provided by the authors was that this value that depends on the resonator Q factor might be significantly improved.

**Figure 6:**
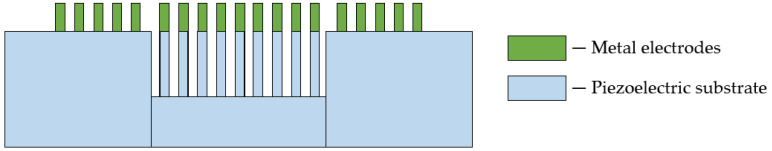
Reflective structure providing the best Q-factor, according to [19].

The next two papers by these researchers were dedicated expressly to the investigation of possibilities for the improvement of the Q factor in the device in question. First, they made an effort to optimize the reflectors’ structure [19]. After considering several approaches, it was shown that the best structure that provided the highest reflection index and, therefore, the best Q-factor, was the one combining open circuit metal electrodes and etched grooves (Figure 6).

**Figure 7:**
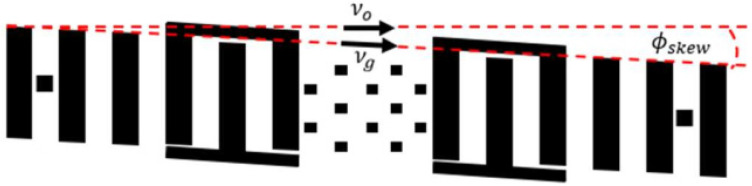
The “skewed” resonator structure from [20].

In 2020, this group of scientists continued their study on the quality factor of SAW resonators [20]. This study demonstrated the potential to minimize losses due to radiation, diffraction, and bulk scattering when exciting resonators on surface acoustic waves with very thick tungsten electrodes (greater than 2% of the acoustic wavelength), necessary to maximize the sensitivity of SAWGs. They formed the walls of masses at a 43-degree angle to minimize scattering from inertial masses. The beam steering effect that was described by the authors is also an important finding. Using both COMSOL FEM simulation and digital holographic microscopy, the authors clearly demonstrated that waves coming from the exciting IDT do not travel exactly normal to the IDT finger edge, but at a slight angle from the expected direction (about ±6.75°). To eliminate this source of losses it was proposed to use a “skewed” resonator (Figure 7).

The research findings have led to the development of SAW devices with the quality factor nearing 15,000, which is more than 14 times higher than that of conventional designs.

**Figure 8:**
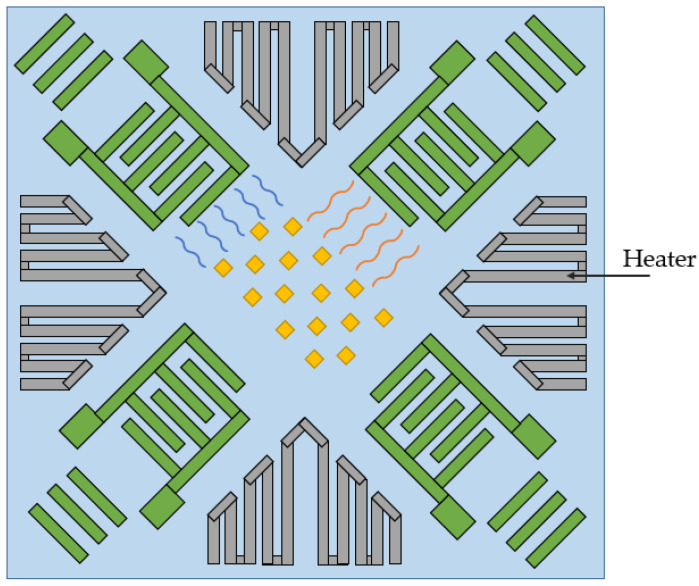
The scheme of the SAWG with ovenization system by Mahmoud Ash. and et al. [21].

Finally, in 2020 the same group proposed a method to improve the aforementioned sensor by integrating an ovenization system into its design [21]. The research was conducted by seamlessly incorporating tungsten serpentine heaters, which were symmetrically arranged around the SAWG, as illustrated in Figure 8. These heaters were operated through a thermal control loop that regulated the SAWG’s temperature at a constant level by continuously tracking the resonance frequency of the SAW.

Their first manufactured prototype demonstrated the ability to minimize temperature fluctuations of the SAWG to less than ±10 µK and stabilize the resonant frequency within the range of ±0.2 ppm. This resulted in the improvement of the angle random walk value up to 1${}^{\circ}/\sqrt{h}$ and bias instability of approx. 25°/h.

Unfortunately, all 4 above-mentioned sources on this topic are rather short conference papers that do not provide sufficient details or a vast discussion. However, the complex work that was made by the authors resulted in numerous useful solutions that are of interest not only in the sphere of designing standing waves-based SAWGs that have definitely been given a new lease of life, but also for other design concepts.

**Figure 9:**
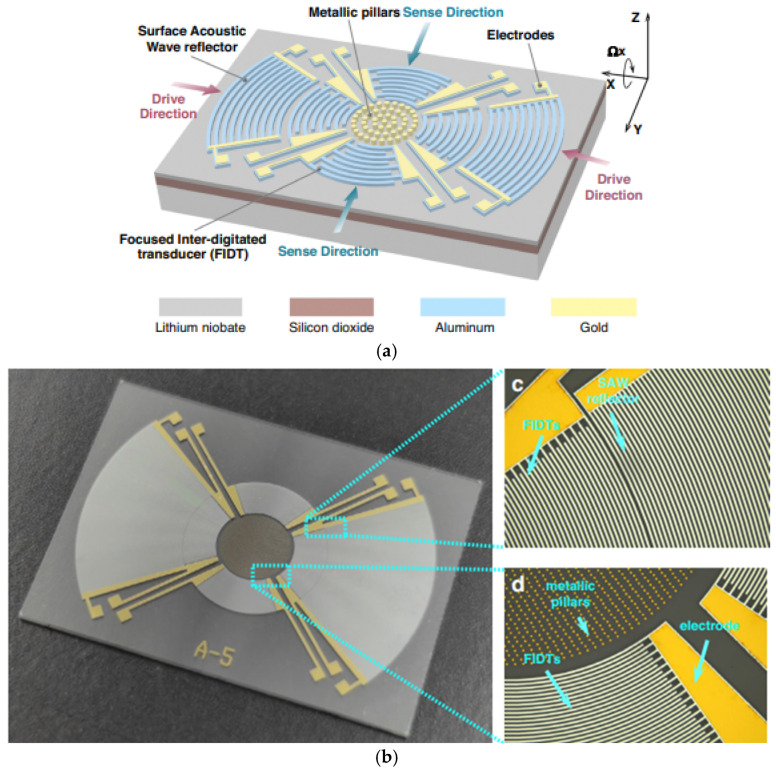
(**a**) The scheme of a standing wave-based SAWG with focused IDTs and (**b**) microphotos of fabricated device (from [23]).

Another study dedicated to the design concept of standing wave based SAWGs and focusing on the optimization of the sensitive element by improving sensitivity and performance was published in 2024 by Tian et al. [23]. Unlike traditional SAW gyroscopes that utilize linear IDTs to generate surface acoustic waves, which leads to beam deflection and energy loss, this research proposes a toroidal shape for IDTs to focus acoustic waves and increase their amplitude (Figure 9).

The experimental results demonstrate that this structure achieves a sensitivity of 1.51 µV/(°/s) and a bias instability of 0.77°/s. This represents a significant improvement of an order of magnitude compared to traditional SAW gyroscopes.

However, this sensor has several drawbacks. First, under high temperatures, the piezoelectric crystal undergoes significant thermal expansion. This affects the material properties and SAW velocity, leading to a drift in the resonant frequency of the SAW resonator and ultimately causing a significant degradation in the gyroscope’s sensitivity. Secondly, another factor is a very weak Coriolis effect in SAW gyroscopes. This results in a weak output signal, severely impacting the sensor’s practical applicability and performance. An evident possibility for further performance improvement would be to combine the ideas proposed in [18,19,20] (e.g., ovenization electrodes, inertial mass form optimization, advanced reflector structures) with the focused electrodes concept from [23].

## 6. New Physical Principles

### 6.1. Co-Existing SAW and BAW

**Figure 10:**
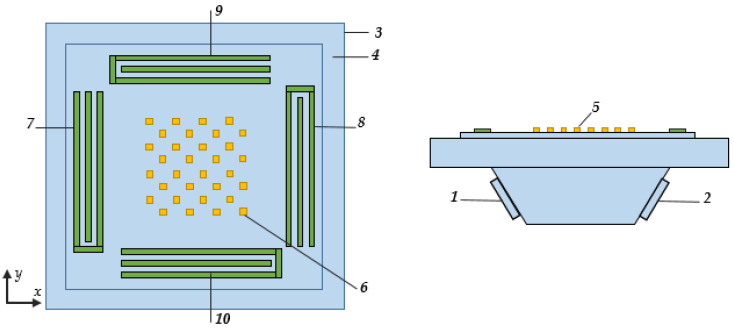
Design of the gyro with bulk acoustic wave excitation proposed in [23].

Several studies have explored gyroscopes utilizing a combination of surface and bulk acoustic waves. One such type was proposed in [24]. The principle of operation for this gyroscope is as follows (Figure 10).

Here, active piezoelectric transducers 1 and 2 generate longitudinal bulk acoustic waves within the supporting base 3. These waves interact with the base’s outer surface 4, exciting surface acoustic waves traveling in opposite directions along the *X*-axis. In region 5, where the regular structure of inertial masses 6 is located, these running waves interfere and create a standing wave with specific distances between the antinodes. When the supporting base 3 rotates around the *X*-axis, the masses moving along the *Z*-axis experience the Coriolis force. This force generates additional SAWs, altering the electrical signals at the outputs of IDTs 7 and 8. The change in signal is proportional to the angular velocity Ω, directed along the *X*-axis. A similar phenomenon occurs when the base rotates around the *Y*-axis, generating signals at the outputs of IDTs 9 and 10.

The authors of the original paper emphasize that one of the unresolved issues encountered in designing the aforementioned SAWGs is the uncertainty regarding how the rotation of the base affects the parameters of the elastic waves propagating within the structure.

In addition, we can see several controversial points in the design:

1. While the main idea is to create a dual-axis sensor, it is obvious that the sensitivity for both axes would be different. This is due to the fact that in the X-direction there are running SAWs induced by the original bulk wave together with waves induced by the Coriolis force, while in the Y-direction there are only waves induced by the Coriolis force.
2. The signal processing scheme is not clear from the description, but should be rather complicated.
3. The signal is expected to be low since a lot of energy flows away from the system as there are no reflectors behind the IDTs.
4. The fabrication process of the device would be very challenging since it requires strict alignment of all its parts. In addition, the complicated shape of the sensing element increases the sensor size, and, probably, reduces its shock resistance.

Surprisingly, this controversial idea has found another implementation in the paper authored by another research group [25]. The main topology of electrodes is taken from the well-known sources [18,22], but the excitation of SAWs comes from the bulk wave, as in [24]. However, instead of adding a trapezoidal projection with two piezoelectric transducers, the authors propose to deposit concentric electrodes on the opposite part of the wafer. Such a solution resolves the energy leakage problems (as there are reflectors in each direction) and simplifies the whole structure. On the other hand, double-sided topology requires a membrane-like clamping of the wafer in its housing and, therefore, reduces the sensor shock-resistance, along with adding the acceleration sensitivity.

It should be noted that the authors not only reported the idea, but fabricated and tested the device. Unfortunately, the effect was registered only along one axis.

In later works [26,27] the same authors reinvented the idea of utilizing both surface and bulk acoustic waves. Here, the electrode topology is still the same as in [18,22], and the operation is also the same. The bulk wave in this case is not used to excite SAWs, but, being induced by the same IDT as SAWs, it is utilized to improve the sensor performance. Normally, the bulk wave coming from IDT is considered a parasitic noise source and suppressed by absorbers on the opposite side of the device. However, the proposed device uses it as a source of additional information. First, as the bulk wave is also sensitive to the rotation rate, its impact can be demodulated from the output signal to enhance the sensitivity. Second, as it has different frequencies compared to the SAW signal, it can be utilized to compensate the temperature drift.

Unfortunately, the paper lacks both a sufficient theoretical description of the Coriolis effect in bulk acoustic waves and detailed instructions on the temperature compensation algorithm. On the other hand, the authors report experimental verification of the proposed principles. The measured uncalibrated scale factor of the gyroscope was 191 µV/(°/s), the angle random walk was 0.028°/√h, while the bias instability was 8°/h, with power consumption of 5.3 mW. Such characteristics make this prototype one of the most promising in the area.

### 6.2. Acousto-Optical Designs

Optical measurement methods are well known for its exclusive precision and resolution. Additionally, integral optical elements possess the same shock resistance as SAW-based sensors. Hence, the idea of incorporating optical measurements to evaluate the effects taking place in SAW devices has always been on the surface. However, the first implementations of acousto-optical gyros (AOGs) occurred only in 2010 [28,29]. The proposed design concepts utilized a light waveguide placed under the SAW delay line. In this case SAWs formed a Bragg grating inside the waveguide and its parameters varied with the rotation velocity. Unfortunately, both papers were quite short and did not provide sufficient details.

**Figure 11:**
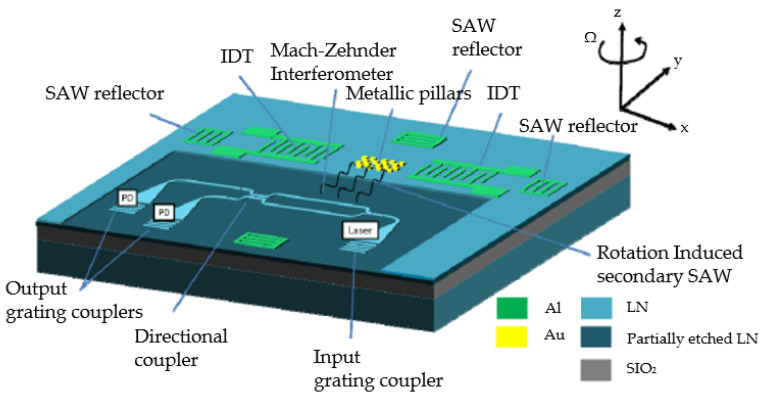
Design of the acousto-optical gyroscope from [30].

The work on the topic was furthered in 2018–2019 by the same group of authors that we have already mentioned in the Optimization section [30,31]. The sensor proposed by them is illustrated in Figure 11.

It utilizes the typical orthogonal structure of SAWGs based on standing SAWs, but without any IDT in the sensing resonator. Instead, there are optical waveguides directed orthogonally to the SAW induced by the Coriolis force (i.e., parallel to the primary “driving” resonator). The sensor was fabricated on the basis of 128° YX-cut LiNbO_3_ on insulator with the whole structure incorporating electrodes and waveguides rotated 45° to the Y crystal direction. This was made, as in the previous work by the same authors [18], to provide equal propagation conditions for the driving and sensing SAWs.

The optical waveguide was proposed in two options: either as the Mach-Zehnder or racetrack interferometer scheme. The SAW induced by rotation ought to change the refractive index in one of the interferometer shoulders, thus making it possible to obtain information about the rotation velocity by measuring the light intensity on the interferometer output. The higher the rotation velocity, the larger the amplitude of the induced SAW is and, therefore, the larger change in the refractive index of the light waveguide will be.

The proposed device was fabricated and tested. The Mach-Zehnder scheme was found to have better performance and showed the scale factor of 48 nV/(°/s). However, this value had approximately ±58% run-to-run variations, which resulted in 60°/√h ARW value. At the same time the bias stability was 1°/s. In conclusion the authors predicted that enhancements made in the fabrication process should lead to a significant increase in the sensitivity and improvement of ARW.

The following paper by the same authors supported these conclusions in 2019 [31]. With the same sensor structure and measurement method, they successfully achieved the scale factor of 0.275 µV/(°/s) and ARW of 4.8°/√h, while simultaneously reducing the device form factor by 2x. It is worth noting that the research also contained the experimental verification of shock resistance, which was found to be not lower than 6000 g. These results clearly show the big potential in designing AOGs, even though they have a rather complicated structure and definitely would be more expensive in production than “pure” SAWGs. The only thing that seems strange in both above-mentioned publications is the mutual direction of rotation velocity and particle vibration. In both papers it is proposed that the device is rotated around the out-of-plane axis (*Z*-axis in Figure 11), and the wave used is of Rayleigh type. Such a combination is similar neither to the scheme used in running waves-based SAWGs (see Section 2.1), nor to the option based on standing waves. The effect would still occur in this configuration since the particles have elliptic trajectories in XZ-plane. However, these ellipses are much wider in Z direction than in X one, making it more efficient to use the larger Z-directed vibration velocity. In this design this component is coincident with the rotation velocity vector, therefore is unable to induce the Coriolis effect. Unless it is just a misprint, it would be interesting to obtain the authors’ comments on this fact, since if they actually meant this, then the question of a sensor cross-sensitivity arises.

**Figure 12:**
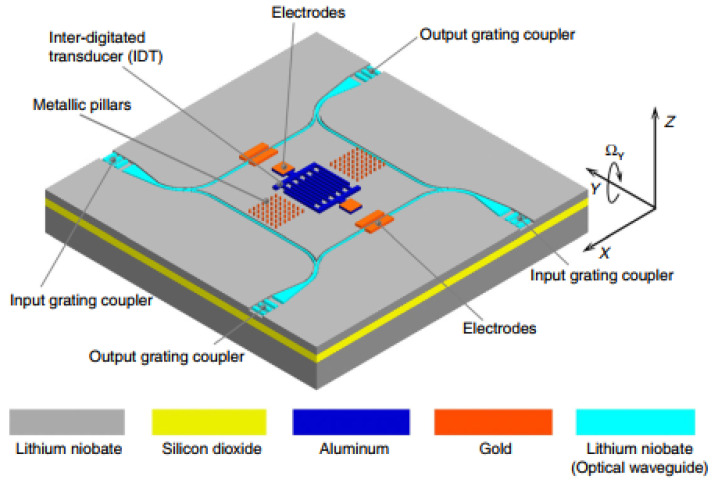
Design of the acousto-optical gyroscope by Lu Tian et al. [32].

In 2022, a group of Chinese scientists suggested another way of AOG realization (Figure 12) [32].

Here, the mechanical stress generated by SAWs is converted into the change in light intensity to be detected using the acousto-optic effect. This progressive wave gyroscope utilizing the acousto-optic effect consists primarily of the following components:

A single IDT for SAW generation.

Metal pillars positioned along the propagation path to magnify the Coriolis effect.

Sputtered electrodes that generate the alternating electric field essential for the device’s functionality.

Optical waveguides etched into the substrate’s surface to facilitate the transmission of optical signals.

**Figure 13:**
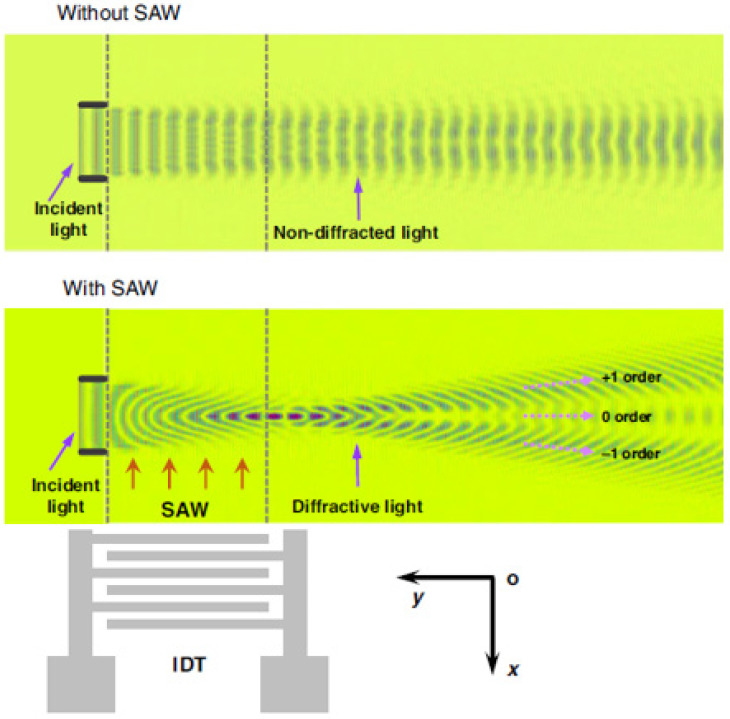
Illustration of Raman–Nath diffraction on SAW (simulation from [32]).

A progressive wave generated by the IDT travels along the *X*-axis. When an out-of-plane rotation Ω is applied along the *Y*-axis, the metal pillars in the wave’s path affect the SAW as shown in Section 2.1. The mechanical stress induced by this SAW alters the refractive index of the optical waveguide. Unlike the design in Figure 11 (proposed in [30,31]), here the optical effect is not the uniform change in the waveguide refractive index, but the formation of a diffraction grating orthogonal to the waveguide (Figure 13).

Waveguides are placed in front of SAWs propagating both in X and –X directions to form a differential scheme and increase the sensitivity. Next, as the authors report, the rotation velocity directed along the *Y*-axis acts through the Coriolis effect and changes the refractive index of the material inside the waveguide, thus changing the diffraction grating parameters. The latter affect the output light intensity.

A simulation of such a sensor in COMSOL Multiphysics showed that the structure sensitivity is 1.8647 (mW/m²)/(rad/s), and the shock resistance reaches 220,000 g—4 times more than it was reported for conventional SAW gyroscopes. Unfortunately, experimental verification of the device operation has not been conducted yet.

The above-described design is definitely novel and utilizes several promising ideas. The topology of the electrodes (ones that are used for the acoustic part) is, probably, as simple as possible—just an IDT and a bunch of metallic pillars. The formation of the diffraction grating instead of the Bragg grating is also an interesting approach. However, the overall design is somewhat controversial.

First, the authors state that it is necessary to position metallic pillars in the antinodes of the wave, but progressive waves do not have any stable nodes or antinodes positions. This might be applicable if additional reflectors are deposited to form a standing wave. It should also result in the increase in the Coriolis effect since a standing wave has a larger amplitude. However, the presented concept does not involve such an option. Also, a running wave would not form a stable but a moving diffraction grating inside the waveguide and the Coriolis effect would change only the velocity of its movement. However, it might be shown that, indeed, in addition to changing the wave propagation velocity, the Coriolis effect also increases the SAW amplitude (which, in its turn, will affect the refractive index in the light waveguide), but this change is almost neglectable. So, the physics of acousto-optic coupling in such an implementation needs to be clarified.

### 6.3. Exotics

First, let us mention a couple of works that are somewhat connected both with gyroscopes and SAW technology, but are not really concepts of SAWGs. In 2018, Pisarenko et al. proposed an intriguing idea to combine a SAW passive ring resonator and a circular SAW resonator (similar to the one used in [33]) to create a Doppler effect between them and utilize such a device to measure the rotation frequency of a shaft or a similar object [34]. However, despite some ambitious innovations, this device is more likely a tachometer than a gyro, so we are not going into details.

Next, in 2024 a group of scientists proposed a quantum gyroscope based on double-mode SAW cavities [35]. In opposite to the previous one, this concept does represent a gyroscope, and a SAW cavity inside it plays an important role. However, the effect that is used to detect the Coriolis force is not the one described in Section 2. All the concepts that we overview in this article and those they are based on, in one way or another, utilize the Coriolis effect acting on particles vibrating in the displacement field of a SAW. Thus, they form the multitude of SAWGs. The concept described in [35] somewhat marginally belongs to this multitude since a SAW cavity is its intrinsic part. However, the nature of the utilized phenomenon is very different and the authors themselves call it a quantum gyroscope rather than a SAWG. Therefore, we have decided to mention this device in case the reader may find some inspiration in it, but will not describe it in details.

**Figure 14:**
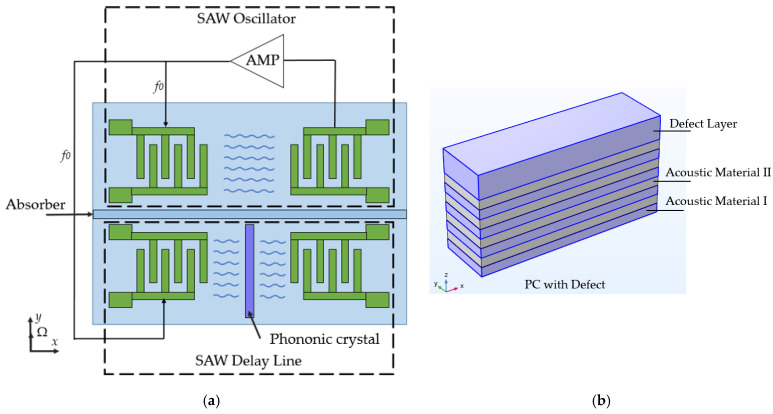
SAW Gyroscope with a phononic crystal: scheme of the device (**a**) and the phononic crystal structure (**b**) [36].

Still, there is at least one concept that is definitely a part of a SAWG multitude but might be considered as a specifically novel finding. It is connected with the usage of a phononic crystal and was proposed in 2021 by a group of Chinese scientists [36]. Figure 14 illustrates the suggested gyroscope structure.

Here, the classic double delay line SAWG concept [1,13] is reorganized in the following way: The first delay line is connected to a feedback amplifier, thus forming a self-oscillating circuit, the output of which is fed to the input IDT in the 2nd delay line. There, a SAW travelling from the input to output IDT hits one or several phononic crystals (PC). The latter is a wall consisting of several pairs of Al and W layers with one additional “defect” Al layer on the top. This structure works as a modulator, which in some range attenuates the SAW amplitude proportional to its frequency. When the SAW frequency reaches the resonant value of the PC, it is mostly absorbed by it. In other cases, its amplitude is attenuated proportionally to the difference of the PC resonant frequency and the SAW frequency in some narrow range. When the SAW frequency is out of this range, it passes the PC almost without any change.

Next, when the rotation velocity is applied along the *Y*-axis, it shifts the frequency of the SAW running in the 1st DL and, consequently, that of the oscillator. Then it additionally shifts the frequency of the SAW running in the 2nd delay line and, finally, the PC converts the overall frequency shift into the amplitude change, which is registered by the output IDT. No experiment was performed by the authors but the simulation showed that the device may have the scale factor of 23.1 mV/(rad/s). However, this characteristic remains linear only in the range of −8 to 8 rad/s. The change in the number of PCs used may increase the linear characteristic range, but at the price of reducing the scale factor. The authors specially emphasized that no metallic dots, which are commonly used to increase the SAWG sensitivity, were used.

In this section, it also is worth mentioning a thesis authored by Kulkarni [37]. He proposes the use of graphene resistors instead of IDTs in classic orthogonal standing wave based SAWGs first proposed by Kurosawa [22] and further considered in many other papers, including the ones reviewed above [18,19,20,21,23]. Such a solution should reduce the mass loading provided by IDTs and significantly reduce their reflection ability. This might be especially helpful in passive wireless devices in order to remove parasitic reflection that may lead to an echo signal (the first concept was proposed in [38], but unfortunately, no other works dedicated to this topic were found in the timespan specified for the review). Additionally, graphene sensing resistors may easily be adjusted in such a way to provide 50 Ohm impedance matching. Unfortunately, the thesis describes mostly the research on the graphene-SAW interaction and the gyroscope is more of an example of its application. Therefore, the thesis does not provide any assumptions concerning the gyro performance improvement.

Finally, we would like to draw attention to the most complex proposal in this review. The patent owned by Amit Lal and Serhat Mehmet Ardanuc [39] offers a variety of both well-known and revolutionary methods to improve the performance of SAWGs, including:

1. Inertial masses (micropillars) to increase the Coriolis effect (as in [13,18,19,20,21,22,23,24,25,26,27,28,29,32]);
2. Perturbation generation through bulk acoustic waves (as in [24,25]);
3. Focusing electrodes for IDT and reflectors (as in [23]);
4. Graphene piezoresistor instead of IDTs (as in [37]);
5. Optical measurement method based on hyperfine transitions in alkaline vapors.

While the first 4 options have already been discussed, the last one deserves a more detailed description. The operation principle is as follows. We deposit a graphene resistor in a SAW resonator. When a SAW reaches it, a current starts to flow inside the graphene and a magnetic field is generated around it. The magnetic field affects alkaline vapors that exist in the closed volume formed by the piezoelectric wafer and the lid. This effect modifies hyperfine transitions of the alkali atom vapor around the graphene resistor. The change is measured optically by utilizing a laser beam that is generated from the backside of the wafer and propagates through the alkali vapor to the lid, which serves as a reflector. Together with the wafer they form a Fabry–Perot resonator. The latter is used to finally perform the optical measurements. The resulting values are compared with the ones from a similar Fabry–Perot resonator but located in the area without a graphene resistor and, therefore, not affected by a magnetic field.

It should be mentioned that all the methods mentioned in [39] are, first of all, not designed to be combined all together, but to be chosen by “a person skilled in the art”. Also, some of them do not strictly apply to a specific SAWG design concept, but might be incorporated in any applicable one. Obviously, such a specific proposal was not accompanied with any experimental evaluations or even theoretical predictions of sensors characteristics.

## 7. The Fabrication Process

Many of the above sources also have a part describing the fabrication process, either the actual one employed to produce the experimental sensor prototype, or just a suggestion for the future. Most of them just repeated the classic photolithography process with small adjustments to meet the specifics of the design. However, we have found two works by the same authors that were fully dedicated to the fabrication process of SAWGs and the process does not involve photolithography [40,41]. The authors proposed to use a laser ablation technique for fast prototyping of SAWG sensing elements. This solution provides a cheap and quick alternative to photolithography and gives the researchers more possibilities to perform field tests of their proposals. Several experiments were described that involved the fabrication of the classic orthogonal Kurosawa topology [22]. It was shown that the method accuracy is suitable for the production of comparatively low-frequency sensitive elements (up to 20 MHz). Taking into account that SAWG sensitivity is usually reverse proportional to the SAW frequency, this limitation does not seem to be a problem.

## 8. Discussion

As can be seen from the above review, although the theoretical basis of SAWGs was first proposed more than 40 years ago, they are still under development. A lot of research groups have made their efforts to bring the idea to life. A big push in this area was made by the American DARPA agency when they launched the PRIGM:AIMS project, which provided funding for many of the works mentioned above [18,19,20,21,25,27,30,31,39]. By showing a real demand from the market, this program stimulated researchers to improve the sensors characteristics. With ARW of 0.028°/√h, and bias instability of 8°/h [27], SAWGs are now approaching the characteristics of conventional MEMS gyros. To compare the characteristics achieved by different devices being reviewed, Table 1 was formed. Here, only the concepts with reported scale factors (simulated or experimental) are considered.

In spite of having several remarkable works in the “Exotics” section, we can state that the majority of works are still based on the same two basic principles proposed by Lee [3] (on running waves) and Kurosawa [22] (on standing waves). From this point of view, probably, the most uncommon concept is the one that utilizes phononic crystals [36]. Even though its main know-how is the use of PCs, the whole structure where DLs are connected in a series instead of forming a differential scheme might be of further interest. It is worth noting here that despite their mutual criticism, both basic concepts are still being developed and show potential applicability, and neither of them might be considered preferrable.

All other findings are mostly either efforts to optimize some elements of well-known devices, or proposals of novel methods of SAW parameter change measurements. The most promising direction is optical measurements [28,29,30,31,32]. Having extremely high resolution and possibility to maintain a solid-state embodiment, such approaches provide plenty of possible schemes, methods and techniques to be chosen and incorporated in the SAWG design.

All in all, today, when plenty of ideas are being generated by researchers from all over the world, the process of SAWG development is more like solving a jigsaw puzzle: we have dozens of pieces (e.g., electrode topologies, measurement methods, material combinations, and special devices such as graphene resistors or phononic crystals) and we need to choose those that fit the best and provide the best picture, i.e., the best sensor performance.

## Figures and Tables

**Table 1:** Comparison of reported experimental or simulation results.

Source	Main Idea	SAW Type	Design Stage	Temperature Sensitivity	Bias Instability, °/s	Scale Factor	Comments
[18,19,20,21]	Orthogonal 45° rotated design with ovenization	Standing	Experimental setup	Ovenization mechanism	0.007	0.959 µV/(°/s)	Papers represent the evolution of the same principle
[23]	Orthogonal design with focused IDTs	Standing	Experimental setup	High sensitivity	0.77	1.51 µV/(°/s)	-
[26,27]	Use of bulk wave mode	Standing	Experimental setup	Temp. compensation by the bulk mode	0.0022	191 µV/(°/s)	Temp. compensation algorithm is not explained
[31]	AOG: Mach-Zehnder interferometer affected by induced SAW	Standing	Experimental setup	Not discussed	2.8 × 10^−4^	0.275 µV/(°/s)	-
[32]	AOG: optical waveguides affected by running SAW	Running	Simulation	Differential scheme	-	1.8647 (mW/m²)/ (rad/s)	Working principle is unclear
[36]	Use of phononic crystals	Running	Simulation	Not discussed	-	23.1 mV/(rad/s)	SF is only for −8…8 rad/s

## Data Availability

No new data were created or analyzed in this study. Data sharing is not applicable to this article.
